# Evaluation of computed tomography settings in the context of visualization and discrimination of low dose injections of a novel liquid soft tissue fiducial marker in head and neck imaging

**DOI:** 10.1186/s12880-021-00689-y

**Published:** 2021-10-27

**Authors:** David Steybe, Philipp Poxleitner, Pit Jacob Voss, Marc Christian Metzger, Rainer Schmelzeisen, Fabian Bamberg, Suam Kim, Maximilian Frederik Russe

**Affiliations:** 1 Department of Oral and Maxillofacial Surgery, Medical Center – University of Freiburg, Faculty of Medicine, Albert-Ludwigs University Freiburg, Hugstetter Str. 55, 79106 Freiburg, Germany; 2 Berta-Ottenstein-Programme for Clinician Scientists, Faculty of Medicine, University of Freiburg, Freiburg, Germany; 3 Department of Diagnostic and Interventional Radiology, Medical Center – University of Freiburg, Faculty of Medicine, University of Freiburg, Freiburg, Germany

**Keywords:** Head and neck cancer, Tumor bed, Fiducial markers, Computed tomography, Dual energy computed tomography

## Abstract

**Background:**

Intraoperative incorporation of radiopaque fiducial markers at the tumor resection surface can provide useful assistance in identifying the tumor bed in postoperative imaging for RT planning and radiological follow-up. Besides titanium clips, iodine containing injectable liquid fiducial markers represent an option that has emerged more recently for this purpose. In this study, marking oral soft tissue resection surfaces, applying low dose injections of a novel Conformité Européenne (CE)-marked liquid fiducial marker based on sucrose acetoisobutyrate (SAIB) and iodinated SAIB (x-SAIB) was investigated.

**Methods:**

Visibility and discriminability of low dose injections of SAIB/x-SAIB (10 µl, 20 µl, 30 µl) were systematically studied at different kV settings used in clinical routine in an ex-vivo porcine mandible model. Transferability of the preclinical results into the clinical setting and applicability of DE-CT were investigated in initial patients.

**Results:**

Markers created by injection volumes as low as 10 µl were visible in CT imaging at all kV settings applied in clinical routine (70–120 kV). An injection volume of 30 µl allowed differentiation from an injection volume of 10 µl. In a total of 118 injections performed in two head and neck cancer patients, markers were clearly visible in 83% and 86% of injections. DE-CT allowed for differentiation between SAIB/x-SAIB markers and other hyperdense structures.

**Conclusions:**

Injection of low doses of SAIB/x-SAIB was found to be a feasible approach to mark oral soft tissue resection surfaces, with injection volumes as low as 10 µl found to be visible at all kV settings applied in clinical routine. With the application of SAIB/x-SAIB reported for tumors of different organs already, mostly applying relatively large volumes for IGRT, this study adds information on the applicability of low dose injections to facilitate identification of the tumor bed in postoperative CT and on performance of the marker at different kV settings used in clinical routine.

## Background

Intraoperative incorporation of radioopaque fiducial markers plays an important role for identification of the tumor bed in postoperative imaging in the context of radiation therapy (RT) planning and radiological follow-up. In breast conserving surgery for example, intraoperative placement of surgical clips in the tumor bed region is considered the current standard to precisely define the former tumor cavity and RT target volume [1–3].

In head and neck cancer, guidelines recommend postoperative RT in case of advanced tumor, close or positive resection margins and cases of cervical lymph node, vascular and/or perineural involvement [4]. In this context, intensity modulated radiation therapy (IMRT) can facilitate delivering targeted RT to the tumor bed, improving locoregional tumor control, while at the same time reducing the dose to the surrounding healthy tissue, resulting in a reduction of early and late RT related complications [5–7]. However, this approach demands highly precise definition of the tumor bed in postoperative imaging to prevent the risk of “marginal miss”, i.e. underdosing in the area of the target volume and to avoid the risk of overdosing in the area of healthy tissue at the same time [7]. Especially in case of free flap soft tissue reconstruction, which is performed frequently in patients with large head and neck tumors, precise delineation of the tumor bed in postoperative medical imaging poses a challenging task due to similar contrast values of native and reconstructed soft tissue [8, 9]. In this context, intraoperative incorporation of titanium ligature clips has been demonstrated to facilitate precise definition of the tumor bed in postsurgical imaging, allowing for an increased accuracy of adjuvant radiation treatment [9, 10].

While metal-based fiducial markers have been in clinical use since many decades, injectable, biocompatible fiducial markers represent a more recent approach with reported advantages compared to metal based markers regarding aspects like artifact level, positional stability and ease of application [11–16]. Recently, it could be demonstrated that amounts as low as 10 µl of a Conformité Européenne (CE) marked iodine-containing liquid fiducial marker (BioXmark®, Nanovi, Kgs. Lyngby, Denmark) are sufficient to create markers in oral soft tissue providing visibility comparable to titanium ligature clips in medical imaging [17]. Moreover, by altering the injected volume, this marker allows for the creation of fiducial markers that can be differentiated by their size visible in postoperative imaging, thus providing the possibility of applying focal injections of higher volumes to delineate areas that require special attention in reporting and RT planning. This could be vascular anastomoses, nerves or regions where a decision against continued resection is made despite intraoperative frozen section analysis not fully ruling out the presence of remaining tumor cells, which might e.g. be the case in areas of delicate anatomical structures.

For RT planning and therapy monitoring purposes in head and neck cancer patients, CT imaging is the modality typically used in clinical routine. Modern CT machines allow examinations of the head and neck region with protocols applying tube voltages between 70 and 150 kV. Extended imaging options have become available by the introduction of Dual-energy (DE) CT. This technique allows for simultaneous imaging at two different energy levels, providing the option for extrapolation of virtual non-contrast (VNC) images, as well as for absorption based characterization of selected molecule weights, that of iodine contained in contrast media for instance [18].

Variability in the size of the markers, created by injection of distinct volumes of BioXmark®, in CT imaging is to be expected at different kV settings due to its iodine content and corresponding k-edge. Moreover, consecutive beam hardening artifacts as well as variability of the distribution of the marker resulting from its initial low viscosity upon injection into soft tissue can be expected to play a role in this context. Thus, the purpose of this study was to quantitatively assess the effect of kV settings, using standard single source technique, on visibility and discriminability of markers resulting from low dose injections of sucrose acetoisobutyrate (SAIB)/iodinated sucrose acetoisobutyrate (x-SAIB). Moreover, it was the aim of this study to evaluate the transferability of these findings on the application of low dose injections of SAIB/x-SAIB into the clinical setting and to assess the potential of DE-CT regarding optimal visualization of the markers by optimized delineation from calcified and metal structures.

## Methods

### Preclinical evaluation of the marker at different kV settings

#### The liquid fiducial marker

The basis of the soft tissue marker investigated in this study (BioXmark®, Nanovi, Kgs. Lyngby, Denmark) is sucrose acetate isobutyrate (SAIB), a chemical structure prepared by esterification of sucrose with acetic and isobutyric anhydride. The major characteristic of this chemical structure is its high viscosity, which can be markedly reduced by adding small amounts of a solvent, e.g. ethanol. This allows for the creation of a product with low initial viscosity, which, due to the diffusion of ethanol, increases rapidly upon injection into soft tissue, resulting in the formation of a hydrophobic, semi-solid, sticky structure. By esterification, it is possible to create iodinated SAIB (x-SAIB), which provides high electron density and thus excellent visibility in X-ray imaging and computed tomography.

The CE-marked medical product BioXmark® is composed of SAIB, iodinated SAIB and ethanol, providing a biocompatible soft tissue marker, which can be injected using thin cannulas and produces positionally stable, radio-opaque fiducial markers.

#### Technical procedure

To provide the basis for the marking of oral soft tissue resection surfaces in the clinical setting, preclinical data on the feasibility of the marking procedure and the performance of the marker in CT imaging at different kV settings was obtained by ex-vivo injection of low doses of SAIB/x-SAIB into the soft tissue of porcine mandible segments. Porcine mandible segments were chosen as they were considered an adequate option to replicate the anatomical structures of the oral cavity (soft tissue, bone, teeth) in CT-imaging. Using a scalpel, the soft tissue in the area of the mandibular ramus was prepared such that it provided a plane surface. Subsequently, 8 to 9 markers were injected per mandible segment, using a consistent pattern to ensure uniform distances between all markers. Injection volumes were 10 µl, 20 µl and 30 µl, which were applied using unit dose injectors (MicroDose, Vlow Medical, Eindhoven, Netherlands) to ensure precise and reproducible injection. Considering the literature available on BioXmark® and its instruction for use, a 25G cannula was used for injection [19]. Aiming at investigating a procedure for marking oral soft tissue resection surfaces, the marker was injected superficially by advancing the needle tip 3–4 mm at a depth of 1–2 mm before injection. To replicate soft tissue defect reconstruction performed in the clinical setting, the soft tissue surface was covered with porcine muscle tissue subsequently to the injection of the SAIB/x-SAIB markers.

#### Acquisition of CT-imaging

CT scans of the porcine mandible segments were performed not earlier than 12 h after injection of SAIB/x-SAIB to allow for reliable efflux of ethanol, as this is a factor potentially affecting the size of the markers visible in imaging. CT imaging was acquired on a clinical scanner (SOMATOM Definition Flash, Siemens Healthineers, Erlangen, Germany) to facilitate the transmission of the results into the clinical setting. In order to analyze the effect of different tube voltage settings on visibility and discriminability of the markers, all injected volumes were scanned at preset voltage values of 70 kV (500 mAs), 100 kV (200 mAs) and 120 kV (120 mAs) with scan protocols used in clinical head and neck imaging**.** The resulting image data was processed using an iterative soft tissue image reconstruction technique (I40s) and a slice thickness of 0.6 mm.

#### Quantification of marker visibility

**Fig. 1:**
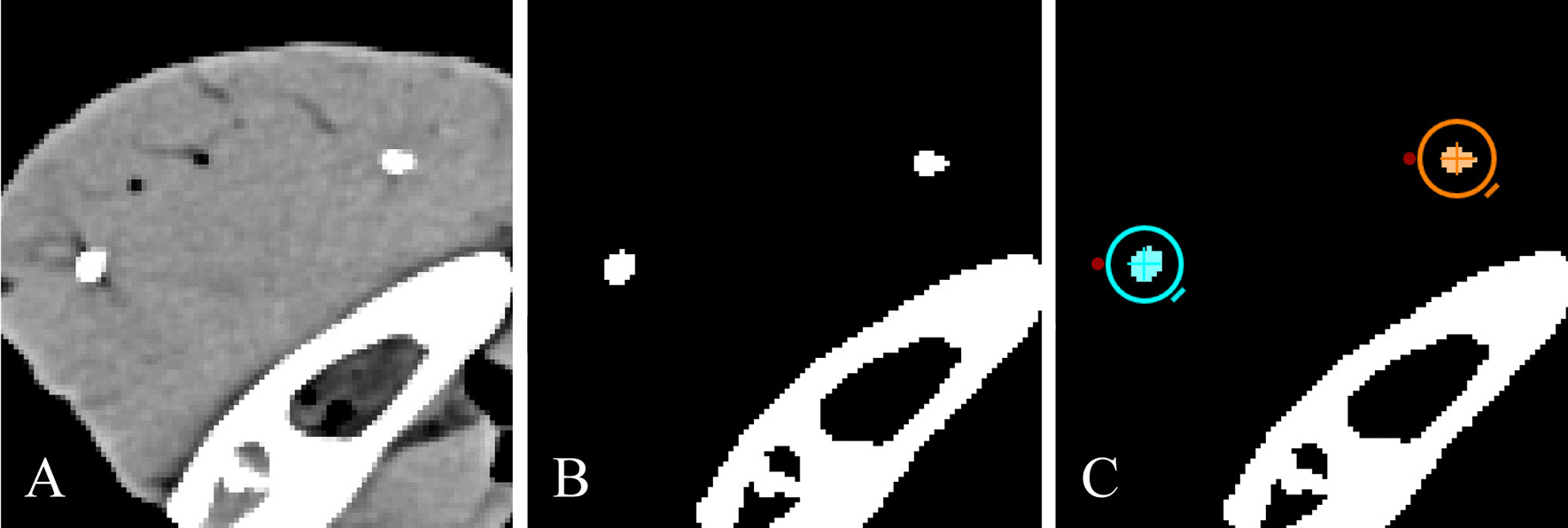
After identification of the markers created by injection of SAIB/x-SAIB in the CT images acquired from the porcine mandible segments (**A**), the lower level of the greyscale window was visually set such that all soft tissue surrounding the markers in the respective CT images was excluded (**B**). Subsequently, the Hounsfield unit value of the lower level was applied as lower threshold to segment the marker volumes by defining a spherical region of interest (ROI) containing the coherent voxels representing each marker (**C**)

In order to facilitate objective quantitative analysis of the effect of different tube voltage settings on visibility and discriminability of the markers in CT imaging, a two-step threshold based segmentation procedure was performed, using the web-based medical imaging platform “Nora Imaging” (www.nora-imaging.com). In a first step, the lower level of the greyscale window was visually set such that all soft tissue surrounding the markers in the respective CT-images was excluded (Fig. 1A, B). In a second step, the Hounsfield unit value of the lower level, which was 140 HU, was applied as lower threshold to segment the marker volumes by defining a spherical region of interest (ROI) containing the coherent voxels representing each marker (Fig. 1C). The volume of each marker determined this way was transferred into a MS Excel (Microsoft, Redmond, Washington, USA) spreadsheet for statistical analysis.

For descriptive statistical analysis, mean volumes, standard deviations, 95% CI and norm intervals (mean ± 1.96*SD) were calculated. Pairwise comparison was performed for statistical analysis of the impact of different kV settings on the segmentable marker volume resulting from a given injection volume of 10 µl, 20 µl or 30 µl.

Moreover, to objectively evaluate whether differentiation between markers created by the injection of 10 µl vs 30 µl was possible at all kV settings investigated in this study, the difference (∆) between the highest segmentable volume resulting from a 10 µl injection and the lowest segmentable volume resulting from a 30 µl injection was calculated. Afterwards, the probability that a 10 µl injection would result in a segmentable volume above a border line (defined as the lower norm interval limit of the segmentable marker volume resulting from an injection volume of 30 µl − ∆/2) was computed, with the results stated as a percentage value. Since the norm interval contains 95% of observations, based on this statistical procedure, a reliable differentiation between the different marker volumes was considered possible for values outside the norm interval (> 95%).

### Clinical evaluation of the marker in single-energy CT imaging

To evaluate the applicability of the marking procedure and transferability of the preclinical results into the clinical setting, a total of 66 (64 × 10 µl; 2 × 30 µl) SAIB/x-SAIB markers were injected at the soft tissue tumor resection surface in a patient undergoing surgical resection of a squamous cell carcinoma, located at the base and lateral margin of the tongue. Injections were performed at the soft tissue resection surface once the results of frozen section analysis were available and surgical removal of the tumor was considered to be adequate. Using unit dose injectors (MicroDose, Vlow Medical, Eindhoven, Netherlands) with 25G cannulas, the needle tip was advanced 3–4 mm at a depth of 1–2 mm before injecting the markers. An injection volume of 10 µl was applied as “basis volume” for the marking of the tumor resection surface. Two regions where, due to the presence of delicate anatomical structures, a decision against continued resection was made despite intraoperative frozen section analysis not fully ruling out the presence of remaining tumor cells were marked by injecting a volume of 30 µl. Subsequently to the injection of the markers, soft tissue defect reconstruction was performed with a rectus muscle flap. Postoperative imaging was performed at 100 kV with a mean of 206 mAs, applying our institution's standard CT protocol for postoperative control in head and neck cancer patients (SOMATOM Definition Force, Siemens Healthineers, Erlangen, Germany).

In order to evaluate whether the results on visibility and discriminability obtained in the preclinical setting were transferrable into a setting based on two-dimensional image analysis, which was considered a prerequisite for the application of the marking procedure in clinical routine, all markers placed in the patient were evaluated by visual slice-by-slice analysis in axial images (slice thickness 0.6 mm; „Bf44 “ reconstruction technique).

### Clinical evaluation of the marker in dual-energy CT imaging

To evaluate the applicability and potential benefits of DE-CT in the context of the proposed marking procedure, DE-CT imaging was performed in a subsequent patient with a total of 52 (48 × 10 µl; 4 × 30 µl) markers placed as described above, after resection of a tumor of the parotid gland and undergoing defect reconstruction with a scapula and latissimus dorsi flap. Imaging was performed on a third-generation Dual Source CT scanner (SOMATOM Definition Force, Siemens Healthineers, Erlangen, Germany) with a protocol applying tube voltages of 80 kV in the A-tube and 150Sn kV in the B-tube, adapted from the proposal by Suntharalingam et al. for DE-CT imaging in head and neck cancer patients [20].

For routine reporting, a mixed-energy image was generated (ratio of 0.6 for A- to B-tube). For marker detection, a mixed-energy image with a slice thickness of 0.6 mm was reconstructed using the same technique as in the single-source protocol. Subsequently, VNC images and iodine maps (3 mm slice thickness), typically used to identify metal structures after surgery, were co-registered to the mixed-energy images to assess whether this could facilitate delineation of SAIB/x-SAIB markers from other hyperdense structures.

## Results

### Preclinical evaluation of the marker at different kV settings

**Fig. 2:**
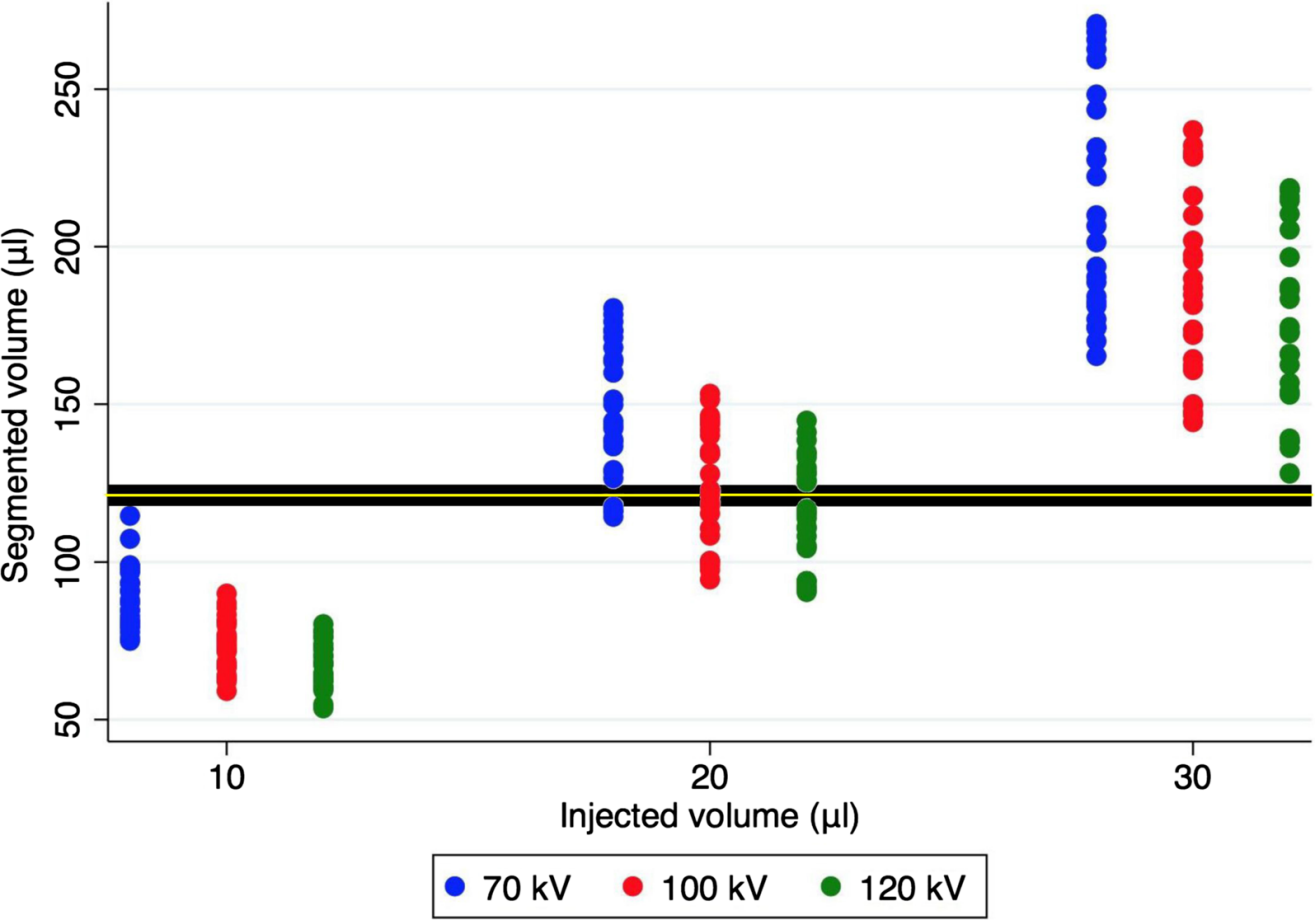
Scatter plot of marker volumes segmented from CT images acquired with tube voltages of 70 kV, 100 kV and 120 kV. The bold horizontal line visualizes the difference (∆) between the highest segmentable volume resulting from a 10 µl injection and the lowest segmentable volume resulting from a 30 µl injection. The yellow horizontal line visualizes the border line applied for statistical evaluation of the differentiability of 10 µl vs 30 µl markers

Creating fiducial markers by performing low dose injections of SAIB/x-SAIB proved to be feasible, with all injections resulting in markers clearly visible in CT-imaging. Placing 25 injections of 10 µl, 20 µl and 30 µl in 9 porcine mandible segments resulted in a total 75 markers and CT-imaging performed with 70 kV, 100 kV and 120 kV thus provided 225 markers the volume of which could be evaluated in this study. An overview of the segmented marker volumes is provided in Table 1 and Fig. 2.

**Table 1:** Overview of segmented marker volumes

Injected volume	10 µl			20 µl			30 µl		
kV	70	100	120	70	100	120	70	100	120
Mean segmented volume (µl)	89.46	73.40	67.48	148.10	125.10	117.14	214.80	188.83	176.48
SD segmented volume	10.14	8.39	7.33	21.71	18.41	16.76	36.87	31.20	29.65
95% CI segmented volume	69.57–109.38	56.94–89.86	53.11–81.86	105.54–190.66	89.00–161.19	84.29–149.99	142.52–287.07	127.67–249.99	118.36–234.59
Ratio segmented volume/injected volume	8.9	7.3	6.7	7.4	6.2	5.8	7.2	6.3	5.9

**Fig. 3:**
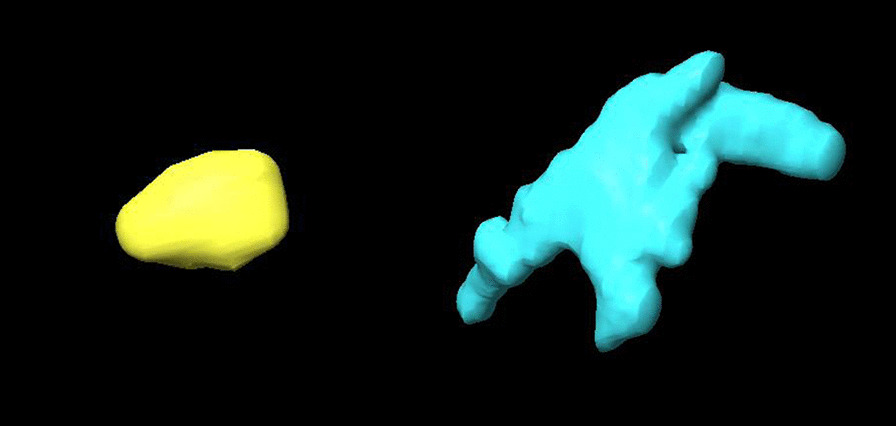
3D reconstruction of markers created by the injection of 10 µl (yellow) and 30 µl (blue) of SAIB/x-SAIB

Evaluating the impact of the injected volume, it could be demonstrated that increasing the injection volume resulted in an increase in the variance of the resulting segmentable volumes (Fig. 2), while at the same time decreasing the ratio between segmented volume and injected volume (Table 1). Moreover, injections of 10 µl of the marker produced relatively homogenous, rounded three-dimensional structures, as opposed to injections of 30 µl, which resulted in markers with a more heterogeneous, multilobular shape (demonstrated in Fig. 3 for representative markers of 10 µl and 30 µl).

Regarding the effect of tube voltage, it could be demonstrated that in general, markers were well discernible with all of the kV settings evaluated, which mirror the spectrum applied in clinical practice. There was a notable negative correlation of segmented volumes with the tube voltage setting applied (Table 1, Fig. 2), explainable by reduced spatial resolution, which corresponded with visual marker impression. These differences between marker volumes (resulting from a given injection volume) segmentable in CT imaging acquired at different kV settings were found to be statistically significant for the 10 µl markers (p < 0.0001 for 100 kV vs 70 kV; p < 0.0001 for 120 kV vs 70 kV; p = 0.019 for 120 kV vs 100 kV). For the injection volume of 20 µl, pairwise comparison revealed p < 0.0001 for 100 kV vs 70 kV; p < 0.0001 for 120 kV vs 70 kV and p = 0.145 for 120 kV vs 100 kV. For the injection volume of 30 µl, pairwise comparison revealed p = 0.006 for 100 kV vs 70 kV; p < 0.0001 for 120 kV vs 70 kV and p = 0.186 for 120 kV vs 100 kV. Thus, low amounts of injected marker are more easily identified and visualized applying lower tube voltage settings, while demarcation improves with higher voltage settings.

Regarding differentiability of the segmentable marker volumes, statistical analysis, performed as described in the materials and methods section, revealed reliable differentiation to be feasible between markers created by the injection of 10 µl and markers created by the injection of 30 µl at all kV settings investigated in this study (values > 95%, which had been defined as cutoff in the statistical procedure).

### Clinical evaluation of the marker in single-energy CT imaging

**Fig. 4:**
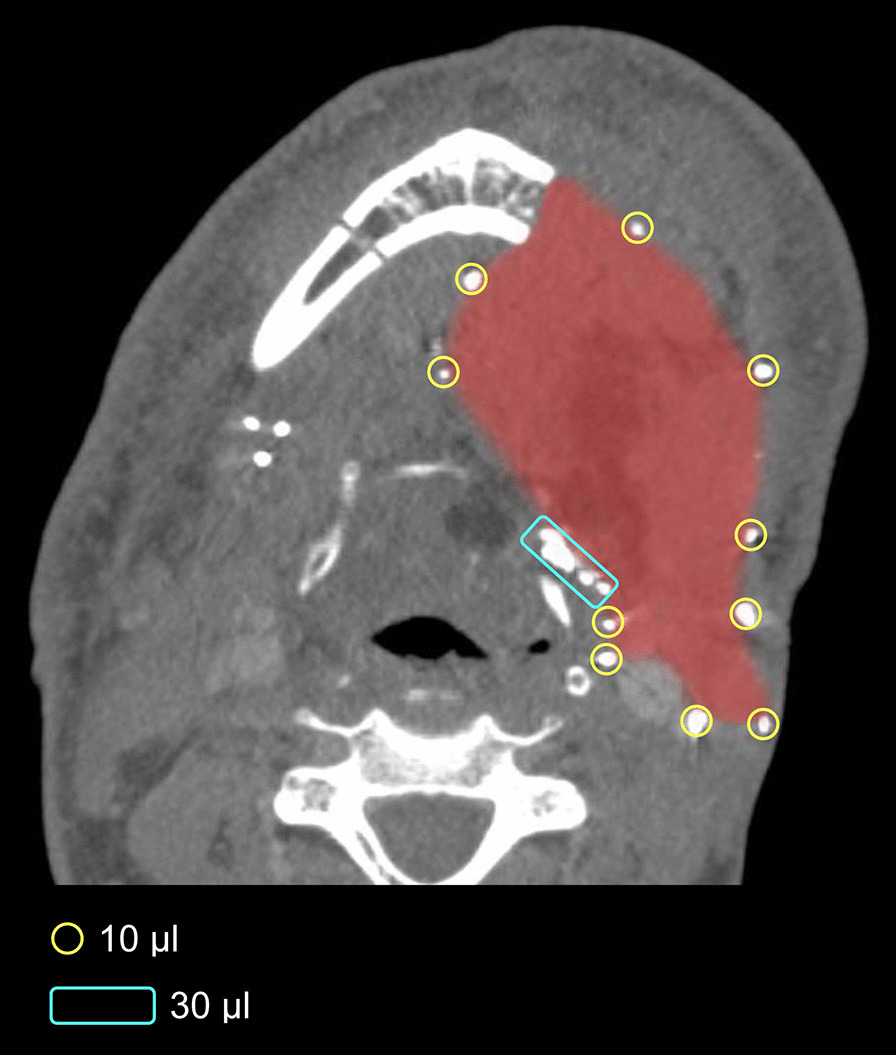
Markers created by injection of 10 µl of SAIB/x-SAIB guide delineation of the tumor resection surface in postoperative CT imaging. Moreover, markers created by the injection of 30 µl of SAIB/x-SAIB can be visually differentiated from the 10 µl markers and thus be applied to mark regions which require special attention in the postoperative setting

Intraoperative injection of 66 SAIB/x-SAIB markers in a patient undergoing surgical resection of a squamous cell carcinoma located at the base and lateral margin of the tongue resulted in 57 markers well identifiable as hyperdense structures in postoperative CT imaging. As was expected from the results of the preclinical investigation, while injections of 10 µl resulted in relatively homogenous, circular markers, injections of 30 µl produced markers that were not only larger but also had a more heterogeneous, non-circular shape, which made them distinguishable from the markers created by injecting the “basis volume” of 10 µl (Fig. 4).

While identification of the markers per se proved to be a straightforward procedure, a difficulty identified in the clinical setting was differentiation of the SAIB/x-SAIB markers from other hyperdense structures present in the same area, e.g. metal clips or calcified structures.

### Clinical evaluation of the marker in dual-energy CT imaging

**Fig. 5:**
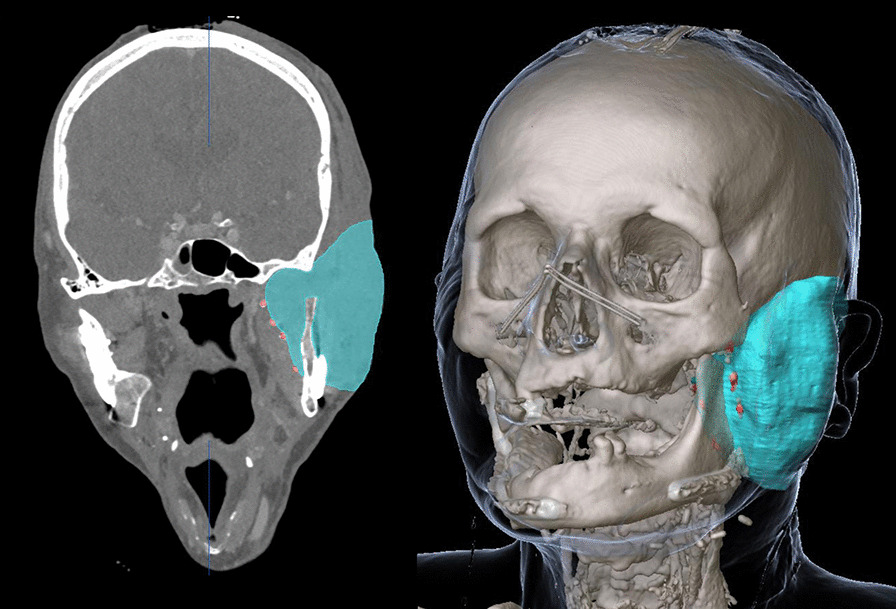
3D reconstruction of the flap volume guided by markers created by the injection of 10 µl of SAIB/x-SAIB

**Fig. 6:**
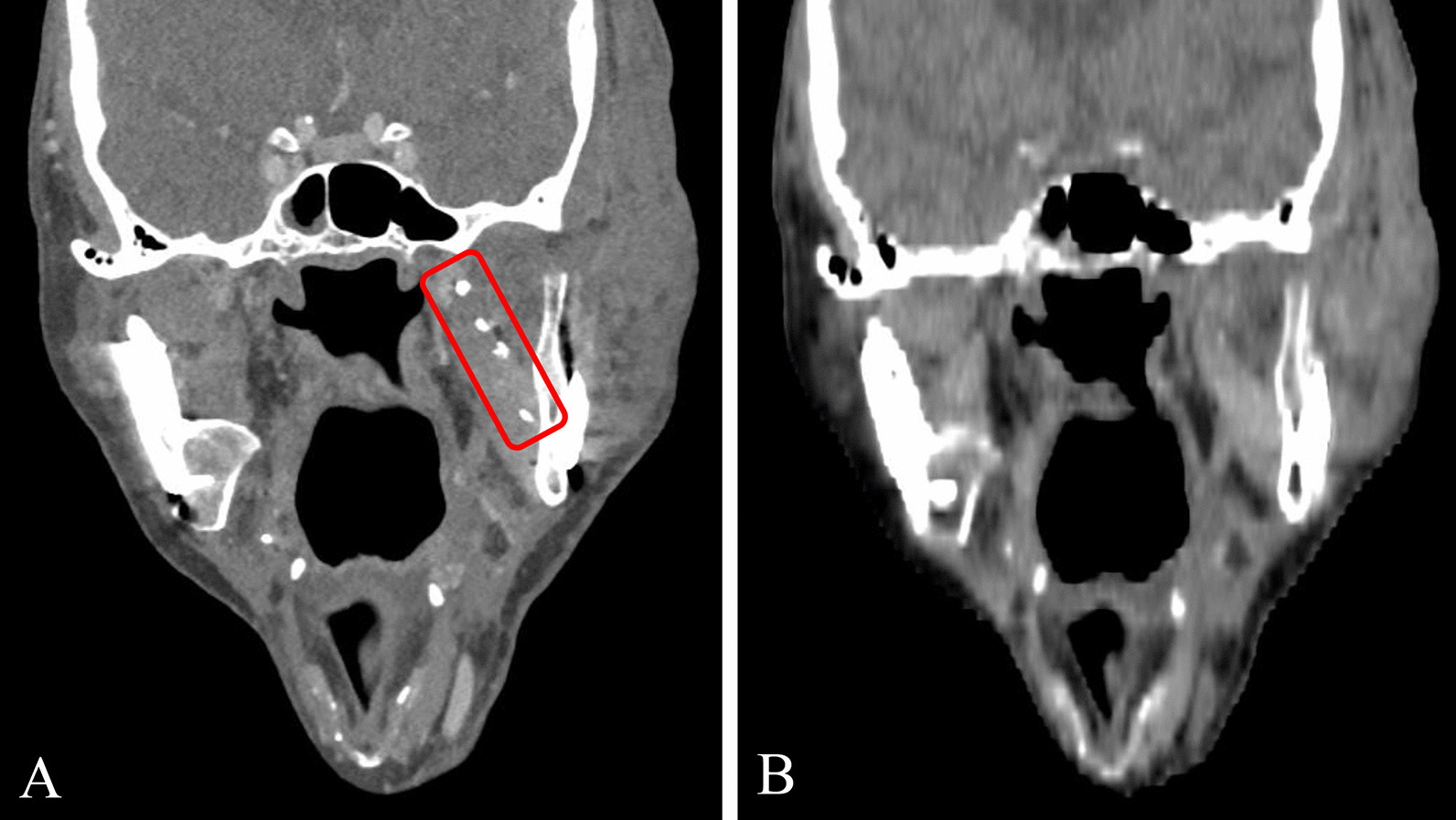
Co-registered view of mixed enery image (**A**) and VNC image (**B**) supports delineation of SAIB/x-SAIB markers from other hyperdense structures (e.g. titanium clips) due to the absence or markedly reduced visibility of the iodine containing SAIB/x-SAIB markers in VNC reconstructions

In DE-CT imaging, which was performed to evaluate the applicability and potential benefits of this imaging technique in the context of the proposed marking procedure, 43 of 52 marker injections resulted in hyperdense structures well identifiable in the mixed energy images and providing the basis for three-dimensional reconstruction of the tumor resection surface/flap volume (Fig. 5). Applying a co-registered view of mixed energy images and VNC images, the absence (in case of 10 µl injections) or markedly reduced (in case of 30 µl injections) visibility of the markers in the VNC reconstruction was supporting the decision for the presence of a SAIB/x-SAIB marker in the mixed energy image. Thus, VNC reconstructions created from the DE-CT images could be demonstrated to provide useful assistance in delineating SAIB/x-SAIB markers from metal clips and calcified structures (Fig. 6). The calculated iodine maps (3 mm slice thickness) also supported the detection of iodine-containing markers but did not support discrimination, as bone and metal objects are often represented as hyperdense structures in these reconstructions as well.

## Discussion

In recent years, promising results have been published from preclinical and clinical investigations into the CE-marked iodine containing liquid fiducial marker BioXmark®, which is based on SAIB/x-SAIB, and it can be expected that liquid fiducial markers will gain increasing relevance in the context of RT planning and delivery as well as in radiological diagnosis and follow-up [13–16, 21–23]. However, while the reports available cover a variety of different tumor sites, to date only little attention has been paid to the application of SAIB/x-SAIB in the head and neck region. Moreover, most of the studies evaluated application of relatively large volumes of the marker for image guided radiotherapy (IGRT), while only little attention has been paid to low dose injections of SAIB/x-SAIB, which might provide a promising option to delineate the tumor bed for precise postoperative reporting, RT planning and RT delivery [17]. As CT is the modality used routinely for head and neck imaging, RT planning and radiological follow up and a range of different kV-settings is applied in clinical routine, the present study investigated different tube voltage settings in the context of low dose injections of SAIB/x-SAIB. As, considering radiation protection, repeated CT scanning for evaluation of the impact of different tube voltage settings on each specific marker would not have been justified in patients, a preclinical investigation was conducted to obtain detailed data that would facilitate quantitative evaluation of this aspect. This part of the study was performed applying an ex-vivo model, as has been reported in other investigations on the preclinical evaluation of SAIB/x-SAIB as well [13, 23]. As good visibility and discriminability of the SAIB/x-SAIB volumes proposed for the marking procedure could be demonstrated in the ex-vivo experiments at all tube current settings investigated, transferability of the concept into the clinical setting was investigated. The results obtained in the first two patients in which the marking procedure has been performed are presented in this paper.

Before forming a gelatinous dimensionally stable structure once injected into soft tissue, the marker has a low viscosity. Thus, variability in the distribution of the marker in soft tissue may result in variability of the marker volumes visible in imaging resulting from a given injection volume [13]. Another major aspect potentially impacting the visible size of the markers in imaging is the tube voltage setting used in postoperative CT imaging of the patient, as tube voltage is known to affect contrast and noise of iodine in CT imaging [24].

In CT imaging of the head and neck region for diagnostic, therapy monitoring and RT planning purposes, single energy CT scans using X-ray voltages between 70 and 120 kV are commonly used. While data on the performance of the marker comparing CT and cone beam computed tomography (CBCT) is available already [12, 17, 23, 25], to date, the effect of tube voltage on the performance of the marker in CT imaging has not been evaluated. Thus, considering the aspects stated above, it was one aim of this study to quantitatively assess the effect of the kV setting applied in postoperative CT imaging on visibility and demarcability of markers created by low dose injection of SAIB/x-SAIB. For this purpose, all porcine mandible segments were scanned at 70 kV, 100 kV and 120 kV. This was done with CT protocols applied in clinical routine, in which each kV setting is associated with a predefined tube current setting. Although tube current can be expected to have an additional impact on contrast and noise in CT imaging [26, 27], we refrained from modifying the preset values in order to avoid impairing the transferability of the preclinical results into the clinical setting.

While all markers could be well detected and demarcated with all CT tube voltage settings applied, volume segmentation results displayed consistent negative correlation to tube voltage setting, which can be explained by voltage-dependent volume distortion [28, 29]. However, despite voltage dependent variations in segmented marker volumes, all markers created by low dose injections of SAIB/x-SAIB, starting at 10 µl, could reliably be identified on postoperative imaging. Moreover, CT-based differentiation between low and higher injection volumes (10 µl vs. 30 µl) was possible at all of the tube voltage settings tested, which mirror the spectrum used in clinical routine.

Having confirmed reliable identification of markers created by the injection of 10 µl of SAIB/x-SAIB to be feasible at all of the tube voltage settings applied in clinical routine in an ex-vivo setting, this volume was used as “basis volume” to mark the tumor resection surface in two patients undergoing resection of extensive head and neck tumors with subsequent soft tissue reconstruction. Intraoperative creation of markers by injecting SAIB/x-SAIB proved to be a straightforward procedure. Evaluating the visibility of the markers in routine postoperative CT imaging revealed 83% and 86% respectively of the SAIB/x-SAIB injections to be clearly visible. This finding is in accordance with the literature available on the clinical application of SAIB/x-SAIB, reporting around 80% of injected markers to be visible in postoperative CT imaging [14, 21–23]. Potential explanations for markers not being visible in postoperative CT might e.g. be manipulation at the resection surface during flap insertion after injection of the marker or injection failure. Another advantage of SAIB/x-SAIB reported in the literature is that markers with varying sizes can be created by altering the injected volume [14]. This could be confirmed in the present study, with differentiation of markers created by the injection of 10 µl and 30 µl demonstrated to be possible via volumetric and 2D image analysis.

While identification of the SAIB/x-SAIB markers proved to be a straightforward procedure, differentiation of the markers from other hyperdense structures present in the area of interest can be difficult. In the past years, DE-CT has been emerging as a promising option to significantly improve lesion detection and tumor delineation in head and neck cancer imaging, with comparable or even reduced radiation exposure [20]. Moreover, by simultaneous imaging at two different energy levels, this technique enables radiation absorption-based tissue characterization. For clinical usage a mixed image based on a weighted mixture of the acquired high and low kV data is calculated. These images are comparable to single energy images of conventional CT-scanners. Additional value can be generated based on the dual energy information. In clinical routine, this provides the option for extrapolation of virtual non-contrast (VNC) images, as well as for absorption based characterization of selected molecule weights, that of iodine contained in contrast media for instance [18]. Evaluation of marker visibility and delineation in DE-CT, performed in a patient with resection of an extensive tumor of the parotid gland and subsequent reconstruction of the mandible and soft tissue, could reveal VNC reconstructions created from the DE-CT images to provide useful assistance in delineating SAIB/x-SAIB markers from metal clips and calcified structures. Thus, DE-CT can be considered a promising option in the context of the proposed marking procedure, as it facilitates reliable differentiation of the SAIB/x-SAIB markers from other hyperdense structures present in the area of interest, e.g. titanium ligature clips, gracile bony structures or calcifications. However, this technology is not a prerequisite for the proposed marking procedure. In single energy CT, evaluation of 3D morphology and/or location, which are specific to the injected markers, surgical clips and e.g. calcifications, is an alternative approach. From our experience, this will facilitate delineation in the majority of cases where an injected marker is located in the same area as other radiopaque structures.

Marking of the tumor resection surface using titanium ligature clips in head and neck cancer patients undergoing surgical tumor resection and subsequent free flap soft tissue reconstruction has been demonstrated to permit reliable delineation of this structure in the context of RT planning. This facilitates significant reduction of the radiation dose administered to the graft while maintaining the boost dose to the planning target volume, including the tumor bed [9, 10]. However, some shortcomings have to be considered when applying titanium clips in this context: Potential migration of metallic clips may reduce the accuracy of the marking procedure and metal induced artifacts might impair their identification on postoperative imaging [13, 15, 23, 30, 31]. Moreover, if placed superficially in the oral/pharyngeal region, there is a risk of clip detachment and subsequent aspiration and from a technical standpoint, placement of the desired number of clips can be impeded by the imperative to avoid intraoperative delay [9, 10].

Evaluating SAIB/x-SAIB as a potential alternative for marking soft tissue resection surfaces, the present study could demonstrate injections as low as 10 µl of the liquid marker to be clearly visible in CT imaging, independent of the tube voltage setting applied. This injection volume results in relatively homogenous, circular markers and allows for a high number of markers to be placed, reducing the distances to be interpolated between the markers and thus increasing the accuracy of the marking procedure. Moreover, low volumes of SAIB/x-SAIB (≤ 50 µl) have been reported to only produce low amounts of artifacts [15, 25], which can be considered beneficial in the context of assessing surrounding structures in postoperative and follow-up imaging. Thus, we propose 10 µl to be used as “basis volume” for the marking of the tumor bed.

Additionally applying injections of 30 µl, which creates markers delineable from the 10 µl markers, results in extended marker capabilities: While 10 µl markers injected with low distances in between can facilitate reliable delineation of the tumor resection surface in postoperative imaging, focal injections of 30 µl can be applied to delineate areas of particular interest in postoperative imaging. Such areas might be regions where a decision against continued resection is made despite intraoperative frozen section analysis not fully ruling out the presence of remaining tumor cells, e.g. in areas of delicate anatomical structures or sensitive structures like microvascular anastomoses or nerves. This approach would provide the basis for special consideration of these specific areas of interest during radiation therapy planning and radiological follow-up. Moreover, taking advantage of the reliable differentiation between SAIB/x-SAIB markers and titanium clips facilitated by the application of DE-CT imaging, reserving the intraoperative application of titanium clips for specific areas of interest would provide a second option for translation of macroscopic and histological information acquired during surgery into postoperative imaging.

To possibly improve marker discernibility and enable finer volume discrimination in future studies, modern high resolution reconstruction techniques or more advanced iodine visualization using multienergy CT approaches could be applied during post processing of CT scans, which remain to be investigated on liquid markers in head and neck surgery and imaging.

## Conclusions

The present study could demonstrate amounts as low as 10 µl of a novel injectable iodine containing fiducial marker to be visible in head and neck CT imaging at all kV settings applied in clinical routine. Based on the findings of this study, intraoperative injection of low doses of this marker can be considered a promising option to facilitate identification of the tumor resection surface in postoperative CT imaging for RT planning and follow-up imaging. Moreover, applying two different volumes of the marker in combination with established titanium clips, intraoperative marking of resection areas is extendable threefold, providing the option for incorporation of intraoperative findings into postoperative imaging.

## Data Availability

All data supporting the findings of this article is included in the article.

## Abbreviations

CBCT: Cone beam computed tomography
CE: Conformité Européenne
CI: Confidence interval
CT: Computed tomography
DE-CT: Dual energy computed tomography
IMRT: Intensity-modulated radiation therapy
kV: Kilovolt
mAs: Milliampere-second
ROI: Region of interest
RT: Radiation therapy
SAIB: Sucrose acetoisobutyrate
SD: Standard deviation
VNC: Virtual non-contrast
VRT: Volume rendering technique
